# Linking pseudo-exfoliation and serum uric acid among cataract patients

**DOI:** 10.6026/973206300211328

**Published:** 2025-06-30

**Authors:** Haneela N.L, Chaitra M.C, Apoorva N., Sasankadhar Reddy

**Affiliations:** 1 Department of Ophthalmology, Sri Devaraj Urs Medical College, Tamaka, Kolar, Karnataka, India

**Keywords:** Pseudo-exfoliation (PXF), serum uric acid (SUA), xanthine oxidase (XO)

## Abstract

Pseudo-exfoliation syndrome (PXF), an age-related condition causing fibrillar deposits in the eye, may involve metabolic factors like uric acid. Therefore, it is of interest to compare blood uric acid levels in 80 cataract patients (over 45 years) at R.L. Jalappa Hospital, Kolar, from January to December 2024, splitting them into 40 with PXF and 40 without, excluding those with secondary PXF, other eye conditions, systemic diseases affecting uric acid, or prior eye surgery and collecting age, sex and uric acid data from medical records. No significant differences were found in mean age (70.83 years for PXF vs. 69.15 years for controls; p=0.332) or sex distribution (p=0.809). However, uric acid was higher in the PXF group (4.513 mg/dL) than controls (3.908 mg/dL; p=0.016), rising with PXF severity: 3.76 mg/dL (mild), 4.49 mg/dL (moderate) and 5.29 mg/dL (severe; p=0.001). Elevated uric acid is linked to PXF presence and severity, suggesting a role in its development, though further studies are needed to confirm clinical significance.

## Background:

Pseudo-exfoliation (PXF) syndrome is an age-related systemic disease that mainly affects the anterior structures of the eye [1]. The condition involves the accumulation of pseudo-exfoliation material, an abnormal fibrillar substance that primarily deposits in the anterior segment of the eye, particularly at the pupillary margin, lens surface and zonules. Recent studies, such as Tanito *et al.* (2012), suggest that oxidative stress contributes to PXF pathogenesis through systemic redox imbalances [2]. Uric Acid (UA) which is one of the most important antioxidants of plasma [3] has been shown to provide up to 65% of total plasma antioxidant capacity [4]. UA can be used as a marker of oxidative stress in hypertension because of the enzyme, Xanthine Oxidase (XO), which catalyzes the reactions of hypoxanthine to xanthine and xanthine to UA and generates one molecule of hydrogen peroxide in each step [5]. In theory, increased activity of XO in PXF can cause hyperuricemia [6]. Supporting this hypothesis, increased adenosine deaminase activity in PXF, which catalyzes an irreversible reaction in purine catabolism, can be an additional factor in increment of UA. On the other hand, increased oxidative stress in PXF might lead to a decrease in uric acid levels, hypouricemia, because it is one of the most important antioxidants of the serum [5]. The role of uric acid levels in pseudo-exfoliation-related oxidative stress has not been clearly studied. Therefore, it is of interest to explore the association between serum uric acid levels and pseudo-exfoliation.

## Materials and Methods:

## Study design:

An observational study.

## Source of data:

Records of patient are who visited Ophthalmology OPD at R. L. Jalappa Hospital, Kolar attached to Sri Devaraj Urs Medical College, Tamaka, Kolar were used.

## Study duration:

January 2024 to December 2024

## Inclusion criteria:

All patients of either sex, more than 45 years diagnosed to have cataract.

## Exclusion criteria: 

[1] Secondary Pseudoexfoliation (due to trauma, surgery, or inflammation)

[2] Other Ocular Diseases (glaucoma [except PXF glaucoma], uveitis)

[3] Systemic Diseases Affecting Uric Acid (gout, chronic kidney disease, uncontrolled diabetes)

[4] Medications Influencing Uric Acid (uric acid-lowering drugs, diuretics, steroids)

[5] History of Ocular Surgery

## Sample size:

A total of 80 cataract patients were included in the study, with 40 patients in each group: Group A comprising patients with pseudoexfoliation and Group B without. Data were collected retrospectively from medical records between January 2024 and December 2024, including demographic data (age, sex), visual acuity, anterior segment findings, PXF material presence/location and intraocular pressure, gonioscopy and serum uric acid levels. PXF severity was graded as mild, moderate, or severe based on the extent of fibrillar material deposition and clinical impact (*e.g.*, zonular instability). Serum uric acid levels were compared between the two groups.

## Statistical methods:

Data will be entered into Microsoft excel data sheet and will be analyzed using SPSS 22 version software. Categorical data will be represented in the form of Frequencies and proportions. Chi-square will be the test of significance. Continuous data will be represented as mean and standard deviation. Independent t test will be the test of significance to identify the mean difference between two groups. P value < 0.05 was considered as statistically significant.

## Statistical software:

MS Excel, SPSS version 22 (IBM SPSS Statistics, Somers NY, USA) was used to analyze data

## Results and Discussion:

A total of 80 cataract patients were included, with 40 in the pseudoexfoliation (PXF) group and 40 in the control group. The mean age of patients in the PXF group was 70.83 ± 7.46 years, while it was 69.15 ± 8.19 years in the control group, with no statistically significant difference (p = 0.332; Figure 1 - see PDF). Gender distribution was comparable between groups (p = 0.809). The mean serum uric acid level was significantly higher in the PXF group (4.513 ± 1.12 mg/dL) compared to controls (3.908 ± 1.08 mg/dL), with a statistically significant difference (p = 0.016). Within the PXF group, patients were further classified by severity: mild (32.5%), moderate (35%) and severe (32.5%). Mean serum uric acid levels increased with severity-3.76 ± 0.98 mg/dL (mild), 4.49 ± 0.80 mg/dL (moderate) and 5.29 ± 1.06 mg/dL (severe)-showing a statistically significant association (p = 0.001). Recent studies, such as Topouzis *et al.* have confirmed a strong correlation between advancing age and PXF prevalence, with higher rates in individuals over 60 years [7]. Similarly, a population-based study in South India by Arvind *et al.* reported that PXF prevalence increases significantly with age, particularly in those aged 70 and above, but found no significant age differences within elderly cohorts when comparing cases and controls [8]. The lack of an age difference in this study suggests that, within an elderly population, age alone may not distinguish PXF cases from controls, consistent with these findings. Gender distribution was comparable between groups, with no statistically significant difference (p=0.809; Table 1 - see PDF). This result is consistent with studies, such as Arnarsson *et al.* in Iceland, which reported no significant sex predilection in PXF prevalence [9]. However, other studies, including Ritch *et al.* have suggested a slight female predominance in certain populations, potentially due to hormonal or genetic factors [10].

The sample size (n=80) may have limited power to detect subtle sex differences, which could explain the absence of a sex effect in this cohort. The mean serum uric acid (SUA) level was significantly higher in the PXF group (4.513 ± 1.12 mg/dL) compared to controls (3.908 ± 1.08 mg/dL), with a statistically significant difference (p=0.016; Figure 2 - see PDF). This finding supports emerging evidence linking elevated SUA with PXF, as hypothesized by previous studies suggesting that hyperuricemia may contribute to oxidative stress and extracellular matrix dysregulation, both implicated in PXF pathogenesis [11]. For instance, Yilmaz *et al.* proposed that oxidative damage to ocular tissues may be a mechanism underlying this association [12]. The observed difference in SUA levels (0.605 mg/dL) suggests a potential metabolic association with PXF, possibly through xanthine oxidase activity, which generates hydrogen peroxide and may exacerbate oxidative stress. However, the modest magnitude of this difference indicates a need for prudent evaluation until larger studies confirm its clinical relevance.

Within the PXF group, patients were classified by severity: mild (32.5%), moderate (35%) and severe (32.5%; Table 2 - see PDF, Figure 3 - see PDF). Mean SUA levels increased with PXF severity-3.76 ± 0.98 mg/dL (mild), 4.49 ± 0.80 mg/dL (moderate) and 5.29 ± 1.06 mg/dL (severe)-showing a statistically significant association (p=0.001; Figure 4 - see PDF). This finding, not previously reported in the cited literature, suggests a dose-response relationship between SUA and PXF severity. The trend aligns with the hypothesis that metabolic factors, including uric acid, may exacerbate the deposition of exfoliative material and associated ocular complications, as proposed by Schlötzer-Schrehardt *et al.* [13]. The association between SUA levels and PXF severity suggests a potential role in monitoring disease progression, though the clinical relevance of these differences requires further validation through longitudinal studies to assess whether elevated SUA predicts PXF progression or can be targeted therapeutically. The study has several limitations. The small sample size (n=80) may limit generalizability and the retrospective design precludes causal inference. Unmeasured confounders, such as dietary purine intake, could influence SUA levels and were not controlled for in this study. Additionally, comparing these results with diverse populations could clarify whether the observed associations are universal or population-specific. Future studies with larger cohorts, prospective designs and adjustment for confounders are needed to confirm the role of SUA in PXF pathogenesis and its potential as a biomarker or therapeutic target.

## Conclusion:

We found no significant differences in age or sex between PXF cases and controls, consistent with recent literature, but identified a significant association between elevated serum uric acid levels and both the presence and severity of PXF. While elevated serum uric acid (SUA) is associated with pseudo-exfoliation syndrome (PXF), the modest difference (0.605 mg/dL) suggests prudent evaluation until larger studies confirm its clinical relevance.

## Financial support and sponsorship:

Nil.
